# Rapid HPLC Method for Determination of Isomaltulose in the Presence of Glucose, Sucrose, and Maltodextrins in Dietary Supplements

**DOI:** 10.3390/foods9091164

**Published:** 2020-08-24

**Authors:** Tomáš Crha, Jiří Pazourek

**Affiliations:** Department of Chemical Drugs, Faculty of Pharmacy, Masaryk University, Palackého 1946/1, CZ-612 00 Brno, Czech Republic; tomascrha@mail.muni.cz

**Keywords:** isomaltulose, Palatinose, maltodextrins, dietary supplements, HILIC, ELSD

## Abstract

This paper presents a rapid HPLC method for the separation of isomaltulose (also known as Palatinose) from other common edible carbohydrates such as sucrose, glucose, and maltodextrins, which are commonly present in food and dietary supplements. This method was applied to determine isomaltulose in selected food supplements for special diets and athletic performance. Due to the selectivity of the separation system, this method can also be used for rapid profiling analysis of mono-, di-, and oligosaccharides in food.

## 1. Introduction

**Figure 1:**
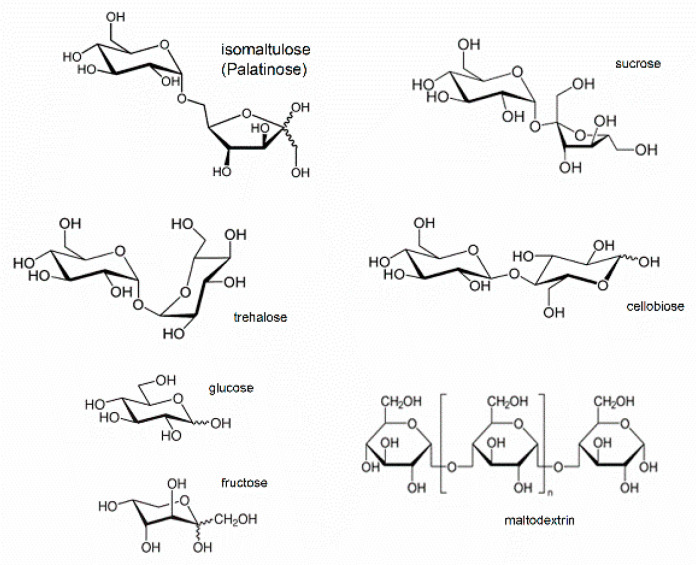
Structures of isomaltulose and other main carbohydrates found in the samples of analyzed food supplements.

Isomaltulose (6-*O*-α-d-glucopyranosyl-d-fructose) or Palatinose is a disaccharide where glucose and fructose are joined by a 1,6-glycosidic bond (Figure 1). It is an isomer of saccharose, which is also the substrate for commercial production by a biotechnological process using bacterial isomerases [1,2,3]. Unlike saccharose, isomaltulose lowers the glycemic index and shows additional health benefits [4,5,6]. Recently, isomaltulose has been shown to improve hyperglycemia in vivo [7] which has brought about related clinical trials [8,9,10]. Isomaltulose can also be found in food and nutrition for diabetics [11].

Currently, isomaltulose is also widely used in fitness supplements for athletes as a long-term source of energy, as this molecule cannot be metabolically broken down (consumed) into glucose and fructose as quickly as sucrose. This has prompted ongoing clinical trials on isomaltulose in dietary supplements for physical performance [12,13,14]. In 2008, the FDA (Food and Drug Administration) added isomaltulose as a substance eligible for the health claim [15] and later the European Food Safety Authority (EFSA) also confirmed the positive health effect of isomaltulose [16,17]. Today, isomaltulose can be found on the market as a substitute for sugar in tooth-friendly chewing gum [18], instant teas (caries to prevent tooth decay) [19], or lifestyle nutrition [20]. Other rapidly metabolized carbohydrates (glucose, fructose) can also be added in commercially available products, as a rapid source of energy; maltodextrins are sometimes present as a long-term energy source [21]. Samples of food and dietary supplements may, of course, contain various of mono-, di-, and oligosaccharides.

In such mixtures of saccharides, determination of isomaltulose is difficult not only because of a limited choice of separation techniques that should separate many isomers of strongly polar compounds, but also due to the absence of good UV-chromophores for their detection.

A common choice is a refractive index detector (RID) which, however, has a low sensitivity and runs can also be time-consuming due to the isocratic elution required. Alternatively, an evaporative light-scattering detector (ELSD) can be advantageously applied for sugar analysis. The ELSD has become a widely utilized detector due to its extraordinary properties; unlike the RID, the ELSD is compatible with gradient elution, which is necessary for chromatographic analysis of complex samples (of natural origin) [22]. With the ELSD, analytes without UV-vis chromophores can be detected, which is especially important for polar compounds. Therefore, the ELSD is quite often connected with Hydrophilic Interaction Liquid Chromatography mode (HILIC) for analytical applications [23,24], including neutral carbohydrates [25,26,27]. Márquez-Sillero et al. compared the ELSD with an alternative detector (corona-charged aerosol detector, CAD) for carbohydrate analysis in food [24]. It is quite important to mention that the ELSD is also sensitive to most common cations and anions; determination of inorganic ions in pharmaceutical formulations by an ELSD has been previously reported [28].

### 1.1. Methods for Saccharides Determination

Methods for determination of carbohydrates are briefly summarized in the following paragraphs. However, polysaccharides analysis is excluded because it is outside the main purpose of this work and related methods can be found elsewhere [29,30].

Traditional methods for mono- and disaccharides determination (sum of saccharides) are volumetry and polarimetry; after the analyte derivatization, photometry or fluorimetry can be applied [31]. A disadvantage of these methods is that they are not selective and cannot be used to determine specific carbohydrates in their mixtures. Selectivity for a given carbohydrate can be achieved by enzymatic methods, where typically glucose, fructose, sucrose, lactose, galactose, maltose, and raffinose are determined, or using separation methods that will be mentioned in the following paragraphs with some of the latest references which may lead a reader to a more comprehensive overview.

High-performance thin-layer chromatography (HPTLC) of oligosaccharides from inulin has been shown by Prošek et al. [32]. Vojvodic et al. recently reported carbohydrate monomer profiling of waste plant biomass using HPTLC [33].

Determination of monosaccharides in complex sugar mixtures by gas chromatography (GC) after derivatization to silyloximes is often applied [34]. GC with UV detection for determination of oligosaccharides has been demonstrated [35], and analysis of sugar content in food products has recently been reported by GC-MS [36].

Gel electrophoresis methods for fluorophore-labeled carbohydrates were reviewed by Jackson [37]. Determination of carbohydrates by capillary electrophoresis with contactless conductivity detection has been utilized for high-energy beverages analysis in 2012 [38]. Recently, microchip electrophoresis application for oligosaccharides has been published [39].

Nowadays, separation of low molecular weight sugars is usually performed by HPLC in two prevailing modes: (i) anion or cation ion-exchange chromatography (on metal-loaded cation exchangers based on a resin or a silica substrate), or (ii) HILIC on amino-propyl modified silica gels.

In the case of ion-exchange chromatography, the analytes are retained between the hydroxyl groups of the saccharides and ionic components of the column stationary phase. In 1980, Mopper et al. reported on the determination of various sugars after separation on the IEC column after complexation with borate, and post-column derivatization with ethylenediamine for fluorometric detection [40]. Recently, Wach et al. [41] compared retention of mono- and disaccharides with high-performance anion-exchange preparative chromatography (HPAEC) on resins in the Ca^2+^–cycle. The method typically uses a strongly basic eluent on ion-exchange resins with pulsed amperometry detection (HPAEC-PAD). HPAEC-PAD with isocratic elution has been recently applied for fermentable short-chain carbohydrates profile (FODMAP) in cereal-product ingredients [42], with gradient elution for mono- and oligosaccharides [43].

Under the HILIC mode, separation methods on amino-propyl stationary phase usually operate with a mobile phase consisting of an excess of acetonitrile (less than 20% water). The excess of acetonitrile is also typical for LC-MS compatible buffers, which are also suitable for detection by ELSD. The buffers may be formate, acetate, or carbonate with an ammonium counter-ion. In 1998 Hernández et al. [44] compared an ELSD with a refractive index detector (RID) for determination of mono- and oligosaccharides in vegetables after separation on amino-propyl stationary phase with mobile phase of acetonitrile-water.

Grembecka et al. [45] developed a method on amino-propyl stationary phase for low concentrations (<0.1 mg/mL) of carbohydrates in food using charged aerosol detector (CAD). A similar column with ELSD for carbohydrate determination in food was reported by Ma et al. [46]. Yoon et al. recently characterized apple quality by various methods, including HPLC for sugar profiling [47].

### 1.2. Methods for Isomaltulose Determination

A recent review on disaccharide determination did not cover isomaltulose [48], perhaps because there is only a limited number of recent papers dealing with the determination of isomaltulose. The articles are briefly described below including retention times of isomaltulose.

Since Japan was the first to introduce Palatinose commercially, the first article on isomaltulose separation and determination appeared there in 1992 [49]. The authors used HPLC on a strong anex column with a direct polarimetric detection. Eluting with 0.5 M borate at 60 °C, they resolved isomaltulose from sucrose, maltose, lactose, and trehalulose in 20 min. The method was applied for determination of isomaltulose in commercially available child foods. In 1996, Yasui et al. reported a HILIC separation and determination of Palatinose in foods on a column with RID [50]. The authors analyzed samples with oligosaccharides; they also demonstrated resolution of maltitol-isomaltulose in 11 min; however, they did not show resolution between sucrose and isomaltulose. Purification of trehalulose from isomaltulose as a by-product was achieved in 81 min by HPLC on a Ca^2+^–catex column [51]. The authors compared HPLC on a strong anion-exchange column where isomaltulose was separated from glucose, fructose, and trehalulose in 11 min. In 2017 Annaratone et al. [52] reported a six-minute analysis (on a 10 cm column) of polar analytes in Belgian endive by HILIC HPLC-ELSD, where isomaltulose served as an internal standard.

Chromatographic separation saccharides and alcoholic sugars, including isomaltulose, in 60 min on zeolites for preparative purposes has been reported [53]. Recently, an article reported a HILIC method with charged aerosol detection for quantitation of carbohydrates in food and beverages; the total analysis time was 30 min, isomaltulose retention time was 20 min [54].

The above-described growing use of isomaltulose in human medicine, as well as in the field of nutritional and physical performance supplements, raises the need for separation methods for the rapid determination of isomaltulose in samples where other common carbohydrates (glucose, sucrose) are present.

This paper presents a fast gradient elution method for determination of isomaltulose by HPLC under Hydrophilic Interaction Liquid Chromatography mode (HILIC) on a polyol stationary phase with ELSD. The retention time of isomaltulose is 6 min, which is a shorter time than in most separation methods published so far.

The presented method (HILIC-ELSD) has several advantages: (i) derivatization of the sample is not necessary, (ii) the polyol stationary phase is able to strongly retain analytes without the need for significant concentrations of either salts or ion-pairing reagents, (iii) saccharides (often isomers) are separated from a mixture (can be identified and determined), (iv) gradient elution necessary for real samples can be easily applied (using MS-compatible buffers), and (v) the buffers are compatible with mass-flow dependent detectors. The applicability of the method is demonstrated on determination of isomaltulose in twenty dietary food supplements (gels and drinks) available on the market.

## 2. Materials and Methods

An HPLC system Dionex Ultimate 3000 (ThermoFisher Scientific, Waltham, MA, USA), with a Varian 380-LC (Varian, Palo Alto, CA, USA) evaporative light-scattering detector was employed. The column was HALO Penta-HILIC (AMT, Wilmington, DE, USA), 150 × 4.1 mm, with particles diameter 2.7 μm. No precolumn was used.

Chemicals were purchased from various sources: acetonitrile of a gradient-HPLC grade, ammonium formate, ammonium bicarbonate, ammonium acetate, formic acid, acetic acid, standards of glucose, isomaltulose, saccharose, cellobiose, trehalose were from Sigma-Aldrich (Merck, Germany). Maltodextrin DE 12-19 was purchased from 4fitness.cz (Brno, Czech Republic).

Samples containing isomaltulose available in the market of the Czech Republic were bought from various producers: Rossmann—Burgwedel, Germany; Heaven Labs—Prague, Czech Republic; Penco—Prague, Czech Republic; Nutrend—Olomouc, Czech Republic; Extrifit—Dolní Újezd, Czech Republic; Amix—Mnichovo Hradiště, Czech Republic; Energikakan—Skeppsbron, Sweden; Vitamin center—Bologna, Italy; for additional information, see Table 1.

### 2.1. The Optimized Separation Method

Stationary phase: HALO Penta-HILIC 4.6 × 150 mm column (AMT), particles 2.7 µm. Mobile phase: 35 mM ammonium formate with acetonitrile. The elution gradient (A = acetonitrile, B = 35 mM ammonium formate, pH = 3.75): 0–5 min 18% B, 5–8 min 18–35% B, 8–10 min 35% B, 10–12 min 35–18% B, 12–15 min 18% B.

Flow rate 2.0 mL/min, thermostat temperature 10 °C, injection volume 4 µL. The detector was an ELSD with the following parameters: gain 2, smooth width 10, evaporator temperature 40 °C, nebulizer temperature 40 °C, nitrogen flow-rate 1 slm (standard liter per minute).

Statistical calculations (standard deviations, relative standard deviation, Grubbs test for outliers) were carried out in MS Excel (Office 2019, Microsoft, Redmond, WA, USA).

## 3. Results and Discussion

Initial experimental conditions for the method optimization were based on recently published papers on study of monosaccharides and disaccharides retention on a polyol stationary phase [25,26,27,55], where a diol column (Lichrosphere100 DIOL, Merck, Darmstadt, Germany) [25,26] was mostly applied, but in this paper we used a HALO PentaHILIC column that showed a better performance [27]. Due to the high affinity towards the stationary phase, a flow rate of 2.0 mL/min could be maintained in all experiments. To identify peaks in chromatograms, retention times were compared with retention times of the individual compounds. Concentrations of standards for the study of experimental conditions (both individual saccharides and in a glucose-isomaltulose-sucrose mixture) were always 1 mg/mL each, 4 μL were always injected. Peak area of isomaltulose and resolution between sucrose and isomaltulose were critical and used as criterions of separation optimization. Experiments were run in triplicate.

### 3.1. Mobile Phase of Acetonitrile-Water (Isocratic Elution)

In HILIC mode, in contrast to reversed phase HPLC, water (or aqueous buffer solution) exhibits the highest elution strength. Firstly, retention behavior of isomaltulose under isocratic elution with a mobile phase containing a mixture of acetonitrile and water was studied. Water content varies in the range of 8–30% (*v*/*v*).

**Figure 2:**
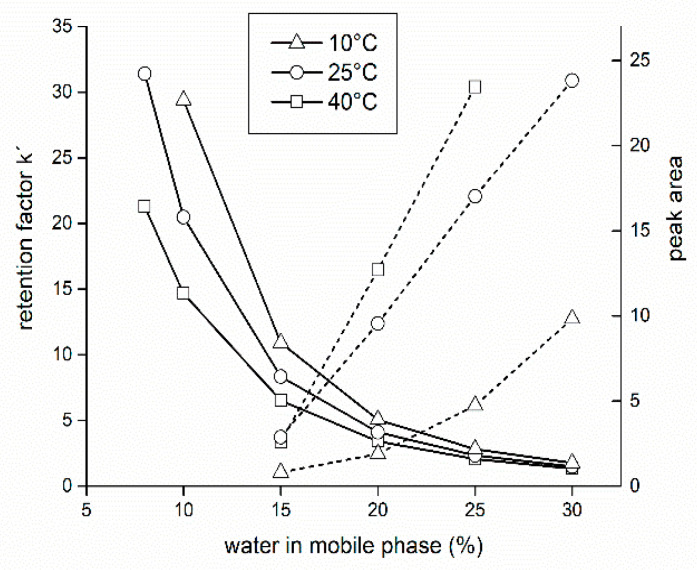
Influence of water content in the mobile phase water–acetonitrile on retention factor *k’* (left *y*-axis, solid lines) and peak area (right *y*-axis, dotted lines), respectively, of isomaltulose. Thermostat temperature was set to 10 °C (open triangles), 25 °C (open circles), and 40 °C (open squares), respectively. Other experimental conditions were: flow-rate 2.0 mL/min, volume injected 4 μL.

As expected, a higher water content in the mobile phase increases the elution force of the mobile phase and thus reduces the retention factor k’ (Figure 2); at water content 30% all the retention factors are practically the same and close to 1. For isomaltulose, the retention factor change was most pronounced at 10 °C (from ≈30 to ≈1.5, see Figure 2, open triangles). To maximize resolution of isomeric analytes in real samples, high retention factors are therefore desirable; typically, they should be higher than 10, which would correspond to a water content of less than 15%.

Comparison of more saccharides retention (glucose-sucrose-isomaltulose, not shown) was in accordance with previous results [25]: it was observed that the retention factor of analytes increases with the number of hydroxyl groups, so that monosaccharides (glucose) elute always before disaccharides (resolution glucose-sucrose > 2) and disaccharides elute before oligosaccharides. Thus, in a test mixture of glucose, saccharose and isomaltulose standards, glucose eluted first, and, at water content > 20%, isomaltulose co-eluted with sucrose; acceptable resolution was achieved for water content < 15% where saccharose eluted before isomaltulose (resolution was approximately 1).

On the other hand, dotted lines (right *y*-axis) in Figure 2 illustrate a dramatic decrease in peak areas as the water content decreases, perhaps caused by a low ionic strength of the mobile phase, which suggests an adsorption of the analytes on the stationary phase, which was previously observed [25,26,27] (adsorption mechanism of separation). Hemström and Irgum [56] have matched arguments supporting partitioning or adsorption mechanism of HILIC from data in the literature. Finding evidence for both models, in conclusions they have agreed with Alpert et al. [57] that “most of the real HILIC separations are in essence multimodal” (a mixed-mode of separation).

To clarify which of the mechanism prevail under our experimental conditions, we carried out an analysis of the retention data based on the following idea: if retention is governed by the partition mechanism, then an empirical equation can describe the process [58]:

(1) $$\log k^{\prime }=\log k{{}^{\prime }}_{w}-S\;\varphi$$

where *φ* is the volume fraction of a stronger solvent (in this case water) in the mobile phase, *S* is the slope when fitted with a linear regression model, and *k’_W_* is the retention factor for the weaker component (organic solvent) only. Thus, a plot of log *k’* vs. *φ* should yield a straight line. If, on the other hand, the retention is due to adsorption then a plot log *k’* vs. log *φ* should yield a straight line, since the best equation describing this situation is the Snyder-Soczewinski equation:

(2) $$\log k^{\prime }=\log k{{}^{\prime }}_{B}-\frac{A_{S}}{n_{w}}\times \log\varphi$$

where log *k’_B_* is the retention factor with the pure solvent *B*, *A_S_* and *n_W_* are the cross-sectional areas occupied by the solute molecule in the surface and solvent molecules, respectively (the ratio *A_S_*/*n_W_* can be considered as a constant).

**Figure 3:**
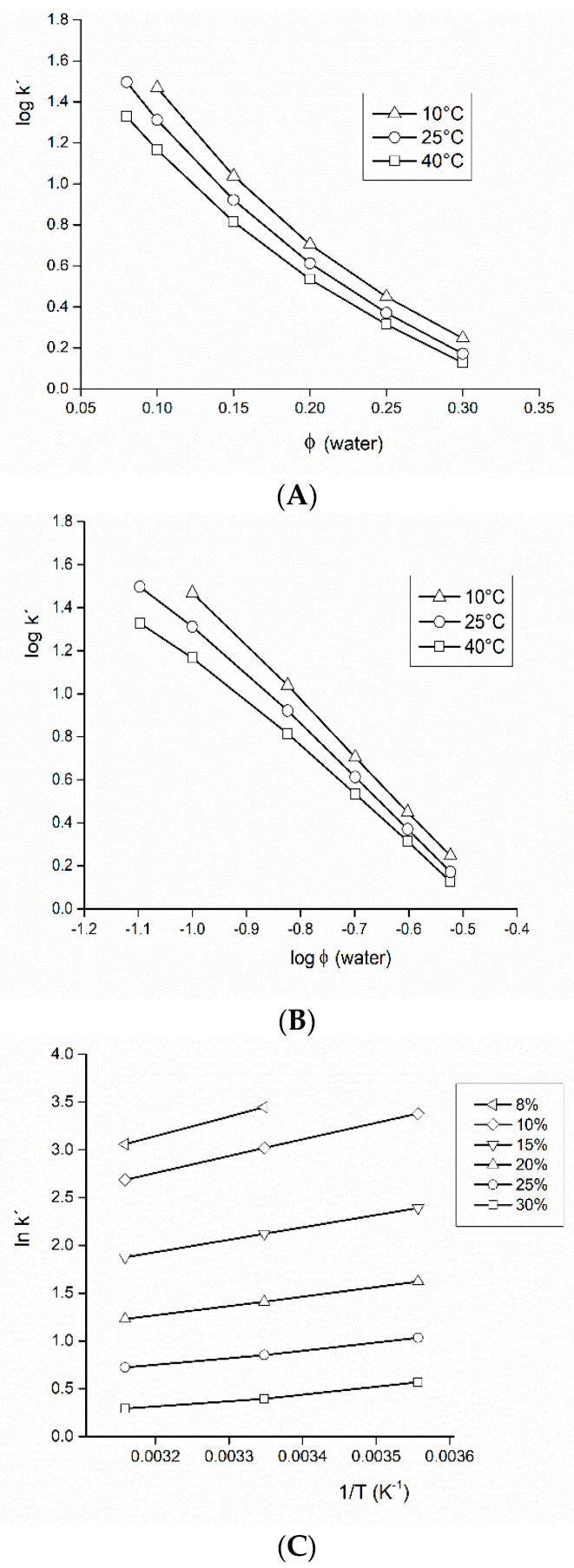
Study of the retention mechanism in the mobile phase water–acetonitrile. Three different mobile phase temperatures (10/25/40 °C) were tested with isomaltulose. From the experimental data, three graphs were constructed: (**A**) presuming the partitioning mechanism-Equation (1); (**B**) presuming the adsorption mechanism-Equation (2); (**C**) van’t Hoff plots-Equation (3). Volume fractions *φ* of water in the mobile phase were 8–30% (*v*/*v*). Other experimental conditions: flow-rate 2.0 mL/min, volume injected 4 µL. For more thermodynamic data see Supplementary data, Table S2.

Since the courses observed on Figure 3A,B, respectively, are not linear, the separation mechanism cannot be considered as either purely controlled by partitioning or adsorption.

Additional information about thermodynamics of the separation process can be obtained from a partitioning chromatography model: if retention is controlled by partitioning between the mobile phase and the immobilized layer of water on the stationary phase, then the relationship between ln *k′* and 1/T should be linear according to van’t Hoff equation:

(3) $$\ln k^{\prime }=-\frac{∆H{}^{\circ}}{RT}+\frac{∆S{}^{\circ}}{R}+\ln\varphi$$

where ∆*H*° is standard retention enthalpy change, ∆*S*° is standard retention entropy change, *R* is the gas constant, *T* is the column absolute temperature.

Van’t Hoff plots in Figure 3C show a decrease in retention as the column temperature is increased. Linearity of the graphs is more pronounced at lower water content (*φ* < 0.15). Negative retention enthalpy values indicated an exothermic process. The enthalpy values derived decreased as the polarity of the mobile phase (water content) decreased, which could indicate the existence of strong specific (secondary) interaction between analytes and functional groups of the stationary phase. Calculated negative standard enthalpy changes were from −0.1 (*φ* = 0.3) to −17 kJ/mol (*φ* = 0.1); so they were maximized (in absolute values) at higher water content, where they exceeded −10 kJ/mol, which is a value typically observed in reversed-phase chromatography [59].

From previous experiments, we can conclude that the water content in the mobile phase and the column temperature are important parameters that affect the retention of polar analytes in HILIC. A low water content and a low temperature would mean a high retention and resolution, and would also produce a dramatic drop in peak area of isomaltulose which is critical for quantitative analysis (limit of detection would be worse). Therefore, a compromise between high retention (high resolution) and sufficient peak area must be chosen. To find better experimental conditions we followed our own experience [27,55]; in the following experiments several ammonium buffers (formate, acetate, bicarbonate) in a mixture with acetonitrile were tested.

### 3.2. Mobile Phase of Acetonitrile-Formate Buffer (Isocratic Elution)

A trend of higher retention due to increasing buffer concentration is related to a hydrophilic partitioning process, because the partitioning model for HILIC assumes the presence of a water-rich liquid layer on the stationary phase. High levels of organic solvent in the mobile phase could cause the salt to be preferentially in the water-rich liquid layer. This would result in an increase in the volume or hydrophilicity of the liquid layer, leading to stronger retentions [60]. However, Alpert [61] has recently demonstrated that well-hydrated counterions serve to promote partitioning of charged solutes into the immobilized aqueous layer in HILIC, while poorly hydrated counterions have an opposite effect. Effects on neutral solutes were more modest; retention times remained unchanged or increased modestly with an increase in concentration of any salt.

In our experiments, water in the mobile phase was replaced for acidic formate buffer (pH adjusted with formic acid). Although dissociation of saccharides negligible is under these conditions (pKa saccharose = 12.6, glucose = 12.3), it affects the adjacent water-rich layer (HILIC mixed-mode).

**Figure 4:**
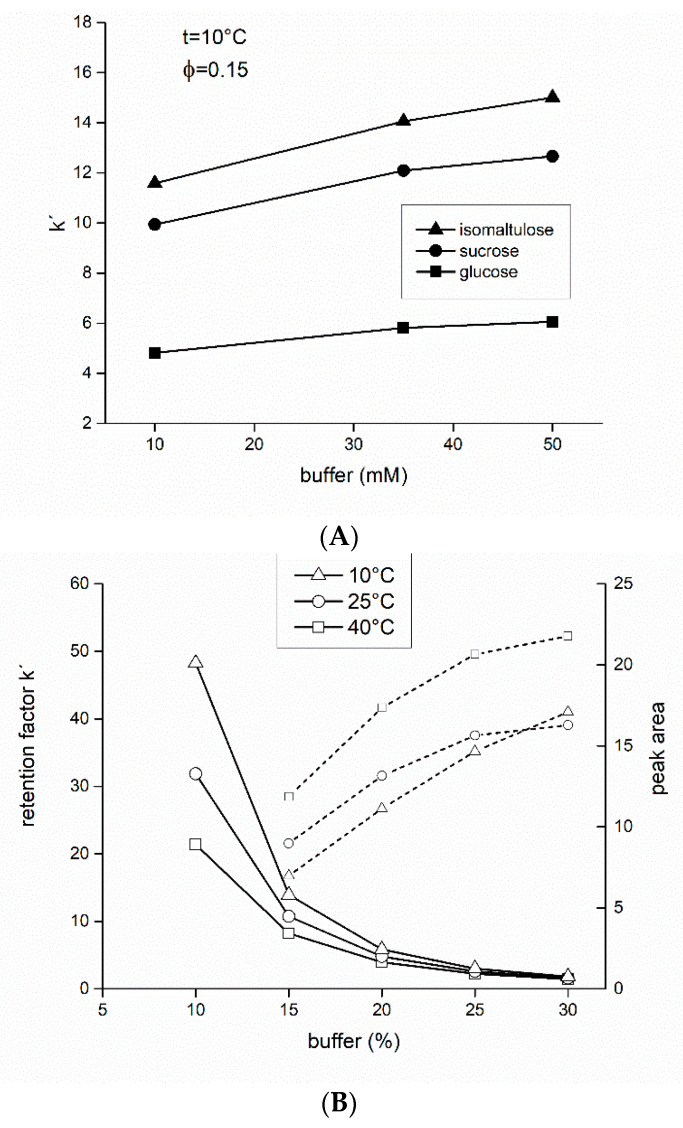
Retention behavior in the mobile phase ammonium formate buffer (pH = 3.75)–acetonitrile. (**A**) Retention factor dependence of three saccharide standards (glucose, sucrose, isomaltulose) on molarity of the formate buffer at its constant volume fraction in the mobile phase *φ* = 0.15, and constant temperature *t* = 10 °C. (**B**) Influence of buffer content on retention factor (left *y*-axis, solid lines) and peak area (right *y*-axis, dotted lines), respectively, of isomaltulose. Thermostat temperatures were set to 10 °C (open triangles), 25 °C (open circles), and 40 °C (open squares), respectively. Other experimental conditions were: flow-rate 2.0 mL/min, volume injected 4 μL, fractions of water in the mobile phase *φ* were 8–30% (*v/v*).

First, the effect of ammonium formate at concentrations 10, 35, and 50 mM, respectively, was investigated on retention of all the standards (glucose, sucrose, isomaltulose) (pH = 3.75, *φ* = 0.15, isocratic elution). An increase in retention time was observed with increasing salt concentration in the buffer (Figure 4A). High buffer concentrations were favorable for higher retention and subsequent resolution. There was no significant difference in retention between the 50 mM and 35 mM buffer, so a concentration of 35 mM was chosen in the following experiments because the retention was high enough to sufficiently resolve the standards (isomaltulose-sucrose).

Next, isomaltulose was tested at three different temperatures and five different buffer volume fractions *φ* (0.08–0.3) (Figure 4B). A comparison of Figure 4B to Figure 2 illustrates that retention factors in the buffered system are higher than in the water–acetonitrile system. The course of the retention factor vs. buffer volume fraction *φ* is similar, but the absolute values of *k’* are higher, especially for low buffer volume fractions (*k’* = 30–50 at 10 °C). Peak areas (comp. dotted lines) at high volume fractions are also comparable.

However, a key finding is that with decreasing volume fractions, the peak areas do not fall so steeply (dotted lines, right axes) in the buffered mobile phase (at *φ* = 0.15 the peak areas are 3–5 times higher), which is important for quantitative analysis (limit of detection, LOD).

Further experiments, analogous to those with the water–acetonitrile mobile phase, aimed to elucidate the separation mechanism in the buffered system. Data obtained were evaluated according to Equations (1), (2), and (3), respectively.

**Figure 5:**
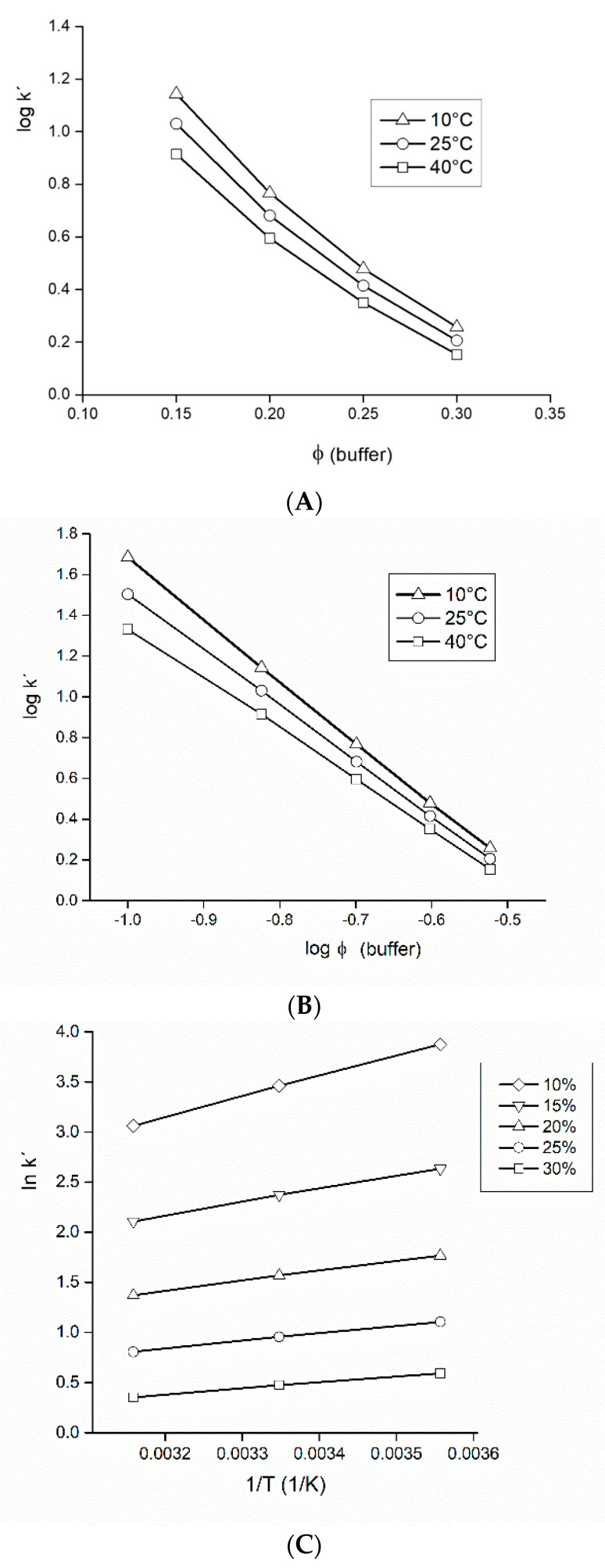
Study of retention mechanism in the mobile phase ammonium formate buffer (pH = 3.75)—acetonitrile. Three different mobile phase temperatures (10/25/40 °C) were tested with isomaltulose. From the experimental data, three graphs were constructed: (**A**) presuming the partitioning mechanism—Equation (1); (**B**) presuming the adsorption mechanism—Equation (2); (**C**) van’t Hoff plots—Equation (3). Volume fractions *φ* of water in the mobile phase were 8–30% (*v*/*v*). Other experimental conditions were: flow-rate 2.0 mL/min, volume injected 4 μL. For more thermodynamic data—see Supplementary Table S2.

A comparison of Figure 5A to Figure 3A (according to Equation (1), a plot log *k’* vs. *φ*) shows a similar non-linear course at all three temperatures. Similarly, courses at temperatures 25 °C and 40 °C, respectively, in Figure 5B, are not linear. A plot log *k’* vs. log *φ* (Figure 5B), however, provided a straight line at 10 °C (open triangles, thick solid line), which favors the adsorption mechanism over the partitioning mechanism.

Despite the limited range of variables tested (five buffer volume fractions *φ*, three buffer concentrations, three standards), we can conclude that in our system the separation mechanism can be considered mixed, controlled by both partitioning and adsorption, which is consistent with the mixed-mode partitioning-surface mechanism from the literature [62,63]. Only at low temperatures do our observations of retention behavior support a predominant adsorption mechanism. Figure 5C (van’t Hoff plots) exhibited similar pattern like graphs in Figure 3C. Calculated negative standard enthalpy changes were in a range from −0.09 (*φ* = 0.3) to −20 kJ/mol (*φ* = 0.1), and were maximized (in absolute values) at higher buffer content, very similar to the acetonitrile-water system.

From the point of view of the separation goal, an important conclusion follows from the comparison of Figure 2 with Figure 4B: the acetonitrile-water mobile phase is not suitable for determining low carbohydrate concentrations in real mixtures where high retentions are required to achieve sufficient resolution. Under these conditions in a non-buffered system, the peak areas are too low. In addition, it is advantageous to use a buffered mobile phase in order to prevent adsorption of contingent components of the matrix (proteins, vitamins, flavors) on the stationary phase.

Therefore, other buffers were tested as components for the mobile phase: ammonium formate at two different pH levels (4.8 and 3.3), ammonium acetate at pH = 5.9, 4.8, and 4.0, respectively, and ammonium bicarbonate (pH = 7.7). The number of theoretical plates, resolution isomaltulose–sucrose and peak area of isomaltulose, respectively, were used as comparison criteria.

For ammonium acetate and ammonium bicarbonate, temperatures of ELSD nebulizer and evaporator had to be raised (60 °C) to stabilize the baseline (bicarbonate) and improve run-to-run repeatability (acetate). However, none of these four buffers provided a better efficiency or resolution for isomaltulose–sucrose. Ammonium formate (pKa = 3.75) exhibited practically the same efficiency (resolution isomaltulose–sucrose) at all the three tested pH levels; at pH = 4.8 slightly higher peak areas were observed, which could lower LOD if this aspect was critical. However, this was not the case (the concentration of isomaltulose in real samples was higher than 1%), so pH = 3.75 was finally selected to maximize the buffer capacity with respect to different matrices of real samples.

**Figure 6:**
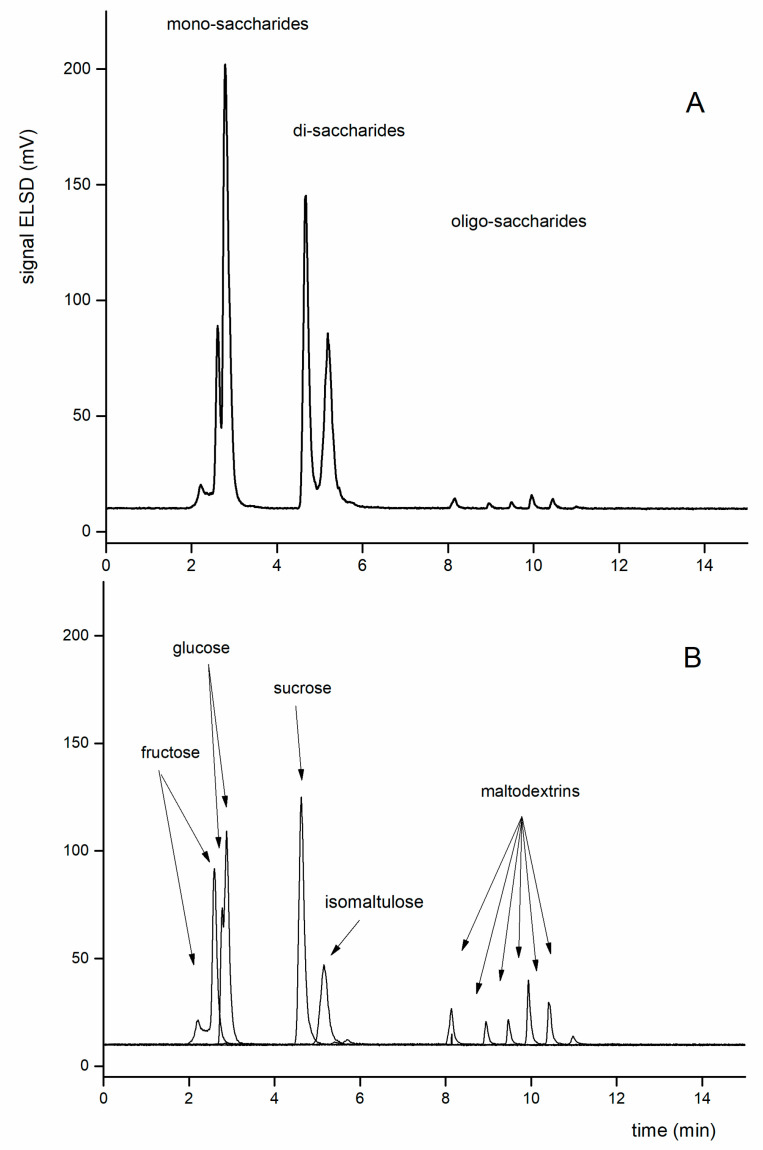
The method performance under optimal conditions (for all the experimental conditions—see text). Comparison of chromatograms of a standard mixture (**A**) and chromatograms of overlapped individual analytes (**B**) under optimized conditions (gradient elution, fructose, glucose, sucrose, isomaltulose, maltodextrins). More peaks of some compound correspond to respective anomers (fructose, glucose—see text) or various numbers of sugar units in oligomers (maltodextrins).

In the following experiments, the mobile phase of the acetonitrile-ammonium formate buffer was always used. To improve resolution, a temperature of 10 °C was chosen. The selectivity of the separation system is so high that is possible to resolve even anomers of saccharides [25]. Therefore, fructose and glucose show multiple signals in chromatograms (see arrows in Figure 6).

### 3.3. Sample Analysis

Based on previous findings, to separate isomaltulose from sucrose, the starting composition of the mobile phase selected was 18% ammonium formate (35 mM) and 82% acetonitrile at 10 °C. Due to the subsequent applications of this method for real samples containing maltodextrins, a gradient elution was applied to elute the oligosaccharide from the separation column. The elution gradient was as follows (A = acetonitrile, B = 35 mM ammonium formate, pH = 3.75): 0–5 min 18% B, 5–8 min 18–35% B, 8–10 min 35% B, 10–12 min 35–18% B, 12–15 min 18% B. For a complete list of experimental conditions see above, Section 2.1. The performance of the method under the optimized conditions is shown in Figure 6.

#### 3.3.1. Calibration

**Figure 7:**
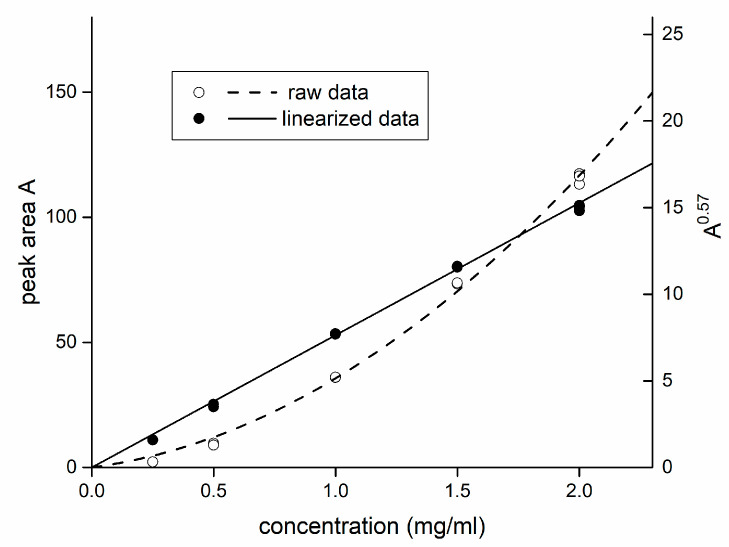
Comparison of a non-linearized (left *y*-axis, open circles, dashed line) and linearized (right *y*-axis, full circles, solid line) calibration curve of isomaltulose under optimized conditions. The calculated exponent (for the transformation procedure, see text) was m = 0.57.

To establish a calibration curve with ELSD, one must consider that a directly obtained calibration curve (peak area vs. analyte concentration) is not linear, and a mathematical transformation should be applied to obtain a linear dependence. A possible approach is to find an exponent m < 1 that the peak area is powered to. Typically, the value lies in the range 0.65–0.80 [27,55]. The exponent can be easily found as a slope of a graph log (concentration) vs. log (peak area). Finally, the linear calibration curve follows an equation with a zero intercept:

(4) $$A^{m}=k\times c$$

where *A* is the peak area, *k* is the calibration slope, *c* is the analyte concentration. Plots before and after the linearization procedure are shown in Figure 7. The calibration range for isomaltulose 0.25–2.0 mg/mL was chosen.

#### 3.3.2. The Method Validation

Validation parameters based on recommendation of ICH Q2 (R1) protocol [64] were obtained within the calibration range 0.25–2.0 mg/mL. It should be noted that due to the non-linear response of ELSD (and linearization procedure applied, see above), any parameter of linearity of the calibration curve (e.g., coefficient of determination R^2^, or quality coefficient QC) is a purpose parameter and is unnecessary to report.

Accuracy (recovery) for isomaltulose was tested with a food supplement Carbo snack gel (green apple flavor, Nutrend, Olomouc, Czech Republic) as a matrix, because its composition is very similar to other running gels and the declared carbohydrate contents were only glucose and oligosaccharides (maltodextrins, xanthan gum) without isomaltulose or other disaccharides. The results from all calibration levels was 97.7–103.8%, which excludes interference of the matrix and suggests that for calibration the method of external standard curve can be utilized. All the validation parameters are summarized in Table 2.

#### 3.3.3. Sample Preparation

A sample preparation procedure should provide a final concentration of isomaltulose at the center of the calibration range ≈ 1 mg/mL.

Samples with a simple matrix (not containing proteins, amino acids or a high amount of interfering additives) were dissolved in distilled water only: typically, 0.1–0.5 g of a gel (or a powder) was weighed and dissolved into a 25 mL volumetric flask, sonicated for 3 min, and then filtrated through a 0.45 μm syringe filter.

Samples with declared proteins or amino acids (Mana drink, Express Energy gel, see Table 1) were mixed with acetonitrile (0.5 g with 1.5 mL of acetonitrile), shaken for 10 min, and centrifuged for 10 min at 13,500 rpm. Then the supernatant was removed and diluted with distilled water.

#### 3.3.4. Determination of Isomaltulose in Samples

The human body is known to rely on carbohydrates as its primary source of fuel because only a limited amount of glycogen can be stored in the muscles. Simple sugars are first absorbed into the bloodstream as glucose, giving a spike in energy. They are then absorbed by active muscles and organs. Physical performance supplements are often classified according to the energy target use. Running gels/running nutrition continue to fuel “quick” energy through monosaccharides, or disaccharides (e.g., isomaltulose) are added for long-term physical performance; the content of oligosaccharides (maltodextrins) in high-energy gels is also important.

The current legislation, the European Parliament Regulation and Council Regulation No. 1169/2011 [65], require food commodity producers to inform consumers about the energy content of foods and beverages. One of the important nutritional parameters is the information about the amount of carbohydrates. According to the definition in the Regulation, “saccharides” are every saccharide metabolized by a human (including polyols), while “sugars” are only mono- and disaccharides (including isomaltulose). It means that long-lasting energy components like oligosaccharides and polysaccharides can be declared as saccharides, but not as sugars.

Isomaltulose was determined in various commercially available products containing isomaltulose and the results were compared with the declared amounts. The samples contained different levels of isomaltulose in the range 0.9–92.8%. Some of the results were partially presented [66].

#### 3.3.5. Beverages

Instant tee Babydream Früchte-Tee (Rossmann, Burgwedel, Germany), with a declared content of 93.9% Palatinose, is in a good agreement (within the method precision) with the determined value of 92.8%, and the chromatogram shows only single peak (see Supplementary data, Figure S1A).

For the Mana non-flavored drink (Heaven Labs, Prague, Czech Republic), 10.1 g saccharides and 2.2 g of sugars, respectively, are declared; the amount of isomaltulose determined was 2.1%. In chromatograms (see Figure S1B) there is a dominant peak of isomaltulose, and several peaks of maltodextrins.

#### 3.3.6. Dietary Supplements for Exercise and Athletic Performance

Among all the supplements tested, the highest content of isomaltulose 32.0% was found in the long-lasting energy gel Enduro snack, apricot flavor (Nutrend, Olomouc, Czech Republic). Although isomaltulose is not declared, it corresponds to 37% of sugars declared (if 5% belongs to glucose). In the chromatogram (green apple—see Figure S1C, apricot—see Figure S2A) only signals of glucose and isomaltulose can be seen.

**Figure 8:**
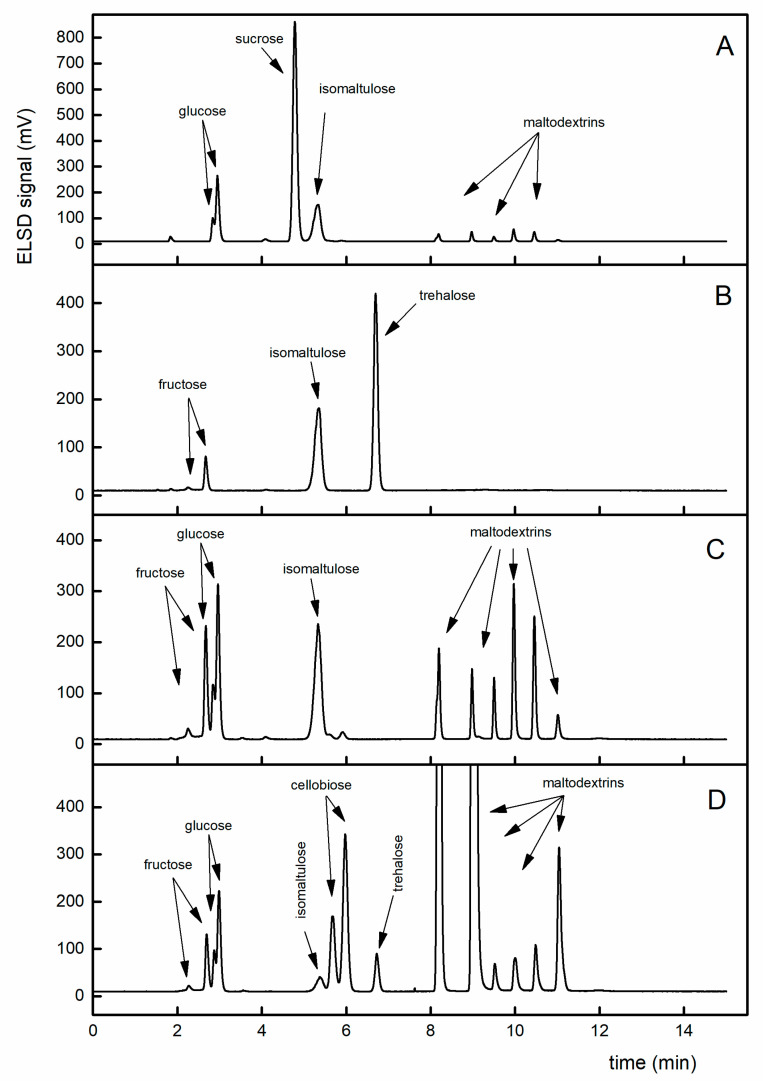
Four examples of chromatograms of the samples from Table 1. The comparison illustrates that the method separates monosaccharides (2–4 min) from disaccharides (5–7 min) and oligosaccharides (8–12 min) and could be used for obtaining a sugar profile (a sugar map). More peaks of a compound (fructose, glucose, cellobiose) correspond to respective anomers. (**A**) Energy drink, orange flavor (Penco, Prague, Czech Republic): declared as 20% isomaltulose, 90% saccharides, and 67% sugars, respectively, determined as 19.0% isomaltulose. (**B**) Vitargo gel, blackcurrant flavor (Energikakan, Skeppsbron, Sweden): declared as 53% saccharides, 45.6% sugars, 18.9% isomaltulose, 18.9% trehalose, determined as 19.3% isomaltulose. (**C**) Energy long trail, raspberry flavor (Penco, Prague, Czech Republic): declared as 68% saccharides, 24% sugars, 10% isomaltulose, 44% maltodextrins, determined as 9.6% isomaltulose; (**D**) Enervit Sport gel, orange flavor (Vitamin center, Bologna, Italy): declared as 80% carbohydrates, 15% sugars, and 1% isomaltulose, determined as 0.9% isomaltulose. For more chromatograms of the samples see Supplementary data, Figures S1–S3.

For the instant energy drink powder (Penco, Prague, Czech Republic), 20% isomaltulose is declared, which is in a good agreement with the amount determined: 19.0% and 21.4% (the same sample in a container and a bag, respectively). The declared content of saccharides is 90% and 67% of sugars, respectively. The chromatogram (see Figure 8A) shows the presence of glucose, sucrose, isomaltulose, and maltodextrins.

Vitargo gel (Energikakan, Skeppsbron, Sweden) exhibits also a high content of isomaltulose, 19.3 and 19.4%, respectively, which is in a good agreement with declared values of 18.9%. In chromatograms, there is a dominating peak of trehalose (see Figure 8B), which is also declared in a high amount (18.9%); a minor component of chromatograms is fructose (without glucose).

Energy gel long trail (Penco, Prague, Czech Republic), 70 g in tubes (or 35 g in packs), various flavors (raspberry, orange, pink grapefruit): the declared value is 10%, determined as 9.6–10.9%. These samples also exhibit fructose, glucose, and maltodextrins on the chromatogram (see Figure S2B), which is in good agreement with the declared sugars, 24% (see Figure 8C).

Express energy gel (Extrifit, Dolní Újezd, Czech Republic) contains a variety of additives including caffein, taurine, tyrosine; the declared value of isomaltulose is 5.0%, determined as 5.3%. The largest peak in the chromatogram (see Figure S2C) is glucose, which corresponds to a declared value of 24%.

The Isogel Carbohydrate Smart Snack juicy orange, green apple (Amix, Mnichovo Hradiště, Czech Republic), 70 mL bag, contains a high signal of fructose and glucose that can be explained by a high declaration of sugars = 42% (declared as isomaltulose, fructose, glucose), 60% of saccharides (maltodextrins and barley starch are declared) (see Figure S3A). The producer does not declare the amount of Palatinose, the amounts determined were 2.4% and 2.2%, resp.

Isogel Energy shock, juicy orange, lemon lime flavor (Amix, Mnichovo Hradiště, Czech Republic), 70 mL bag, contains the greatest number of peaks on the chromatograms among our samples, caused by many declared additives like amino acids, caffein, xanthan gum (a polysaccharide), and starch, which corresponds to a high content of saccharides (60%) (see Figure S3B). The producer does not declare the amount of Palatinose; the determined amounts were 2.5% and 2.4%, resp.

Rock’s! energy gel, lemon lime flavor (Amix, Mnichovo Hradiště, Czech Republic), 32 g bag, was very similar to Isogel Energy Shock (Amix, Mnichovo Hradiště, Czech Republic), with an even higher declared content of saccharides (78%) (xanthan gum), with no amino acids or caffeine. The extremely high signal of fructose and glucose can be explained by the high declaration of sugars = 47% (fructose, glucose, and isomaltulose are declared, see Figure S3C). The producer does not declare the amount of isomaltulose; the determined amount was 2.0%.

Enervit Sport gel (Vitamin Center, Bologna, Italy) has a unique declaration of fructose syrup which is a mixture of glucose and fructose (declared as 2%). Trehalose was also present (declared as 1%) and relatively high signals of maltodextrins. The declared content of isomaltulose is 1%; the amount found is 0.9% (see Figure 8D).

## 4. Conclusions

A rapid separation method was developed and applied for determination of isomaltulose in the presence of sucrose, glucose, and oligosaccharides. The application of the method to commercially available samples of beverages and dietary supplements for athletic performance mostly confirmed declared amounts of isomaltulose within the experimental precision (≈2%). If there was amount of isomaltulose not declared, the determined value was lower than a declared amount of “sugars”.

This method can be used to determine isomaltulose (or other saccharides) in food supplements of so-called healthy food, and also in food supplements for physical exercise and athletic performance, the assortment of which is currently under development; eventually to determine isomaltulose as a potential drug against diabetes. The method could be also used for sugar profiling of food (after appropriate sample preparation). Baseline separation of sucrose and isomaltulose would allow this method to be used to control or monitor adulteration of isomaltulose.

## Figures and Tables

**Table 1:** Analyzed samples. The columns contain the type of a sample, its commercial name, flavor, package, type and producer (see Materials and Methods for the producer’s origins); the last two columns contain declared and determined amount of isomaltulose. The method precision was 1.1–2.4%, respectively. For additional information see Supplementary data, Table S1.

Type of Sample	Commercial Name, Flavor	Producer, Package Type	Isomaltulose (or Sugars) Declared	Isomaltulose Determined
**Beverages**				
instant tee (granulate)	Babydream Früchte-Tee	Rossmann container 190 g	93.9%	92.8%
complete-nutrition food (drink) ***	Mana non-flavored drink	Heaven labs box 330 mL	2.2% (sugars)	2.1%
**Dietary Supplements for Exercise and Athletic Performance**				
Energy gel	Enduro snack green apple	Nutrend tube 75 g	37.0% (sugars)	30.1%
Energy gel	Enduro snack apricot	Nutrend tube 75 g	37.0% (sugars)	32.0%
Energy drink (powder)	Energy drink orange	Penco container 900 g	20.0%	19.0% (Figure 8A)
Energy drink (powder)	Energy drink orange	Penco bag 30 g	20.0%	21.4%
Gel	Vitargo gel black currant	Energikakan bag 45 g	18.9%	19.4% (Figure 8B)
Gel	Vitargo gel watermelon	Energikakan bag 45 g	18.9%	19.3%
Gel	Energy gel long trail raspberry	Penco tube 70 g	10.0%	10.5% (Figure 8C)
Gel	Energy gel long trail orange	Penco tube 70 g	10.0%	10.9%
Gel	Energy gel long trail lemon	Penco bag 35 g	10.0%	9.8%
Gel	Energy gel long trail orange	Penco bag 35 g	10.0%	9.6%
Gel	Energy gel long trail pink grapefruit	Penco bag 35 g	10.0%	9.9%
Gel ***	Express Energy Gel	Extrifit capped bag 80 g	5.0%	5.3%
Gel	IsoGel Smart Snack juicy orange	Amix capped bag 70 mL	42% (sugars)	2.4%
Gel	IsoGel Smart Snack green apple	Amix capped bag 70 mL	42% (sugars)	2.2%
Gel	IsoGel Energy Shock juicy orange	Amix capped bag 70 mL	46.6% (sugars)	2.5%
Gel	IsoGel Energy Shocklemon lime	Amix capped bag 70 mL	46.6% (sugars)	2.4%
Gel	Rock’s! energy gel lemon lime	Amix bag 32 g	46.6% (sugars)	2.0%
Gel	Enervit Sport gel, orange	Vitamin Center bag 25 mL	1.0%	0.9% (Figure 8D)

*** Different sample preparation, see text.

**Table 2:** Validation parameters of the presented method. For details see text.

Calibration Range	0.25–2.0 mg/mL
Accuracy/Recovery (0.25–2.0 mg/mL)	97.7–103.8%
Repeatability (retention time)	0.2%
Repeatability (peak area)	1.1%
Intermediate precision (peak area)	2.4%
Limit of detection, LOD (S/N = 3)	20 mg/L
Limit of quantification, LOQ	66 mg/L

## Supplementary Materials

The following figures and tables are available online at https://www.mdpi.com/2304-8158/9/9/1164/s1, Figure S1: Chromatograms of the rest of the samples, Figure S2: Chromatograms of the rest of the samples, Figure S3: Chromatograms of the rest of the samples, Table S1: More information on the samples–links to producers’ web pages, Table S2: Thermodynamic data of isomaltulose in the two compared HILIC systems.
